# Urbilaterian origin of paralogous GnRH and corazonin neuropeptide signalling pathways

**DOI:** 10.1038/srep28788

**Published:** 2016-06-28

**Authors:** Shi Tian, Meet Zandawala, Isabel Beets, Esra Baytemur, Susan E. Slade, James H. Scrivens, Maurice R. Elphick

**Affiliations:** 1 Queen Mary University of London, School of Biological & Chemical Sciences, Mile End Road, London, E1 4NS, UK; 2 Functional Genomics and Proteomics Group, Department of Biology, KU Leuven, Leuven, Belgium; 3 Waters/Warwick Centre for BioMedical Mass Spectrometry and Proteomics, School of Life Sciences, University of Warwick, Coventry, CV4 7AL, UK

## Abstract

Gonadotropin-releasing hormone (GnRH) is a key regulator of reproductive maturation in humans and other vertebrates. Homologs of GnRH and its cognate receptor have been identified in invertebrates–for example, the adipokinetic hormone (AKH) and corazonin (CRZ) neuropeptide pathways in arthropods. However, the precise evolutionary relationships and origins of these signalling systems remain unknown. Here we have addressed this issue with the first identification of both GnRH-type and CRZ-type signalling systems in a deuterostome–the echinoderm (starfish) *Asterias rubens*. We have identified a GnRH-like neuropeptide (pQIHYKNPGWGPG-NH_2_) that specifically activates an *A. rubens* GnRH-type receptor and a novel neuropeptide (HNTFTMGGQNRWKAG-NH_2_) that specifically activates an *A. rubens* CRZ-type receptor. With the discovery of these ligand-receptor pairs, we demonstrate that the vertebrate/deuterostomian GnRH-type and the protostomian AKH systems are orthologous and the origin of a paralogous CRZ-type signalling system can be traced to the common ancestor of the Bilateria (Urbilateria).

Neuropeptides are important regulators of physiological processes and behaviour in humans and other animals^1^,^2^. One of the most widely known and well-studied neuropeptide signalling pathways is the gonadotropin-releasing hormone (GnRH) system, which controls reproductive maturation and function in humans and other vertebrates. Thus, GnRH stimulates release of the gonadotropic hormones luteinizing hormone (LH) and follicle-stimulating hormone (FSH) from the pituitary gland^3^,^4^.

Homologs of GnRH have been identified in invertebrates, including adipokinetic hormone (AKH), corazonin (CRZ) and AKH/CRZ-related peptide (ACP) in arthropods. AKH is a lipid-mobilizing hormone in insects that is released during flight and other energy utilizing activities^5^. CRZ was discovered on account of its stimulatory effect on heart rate in cockroaches^6^ but it also has other functions that range from initiating ecdysis in moths to triggering gregarization-associated dark-pigmentation in locusts^7^,^8^. The recently discovered ACP signalling system is a paralog of the AKH system that arose in a common ancestor of arthropods^9^,^10^, but its functions remain unclear^11^.

Although GnRH-related neuropeptides have been studied extensively in chordates and arthropods, their evolutionary relationships are a matter of debate, largely due to lack of information from other phyla. It is well-established that AKH, ACP, CRZ and GnRH form a superfamily and that protostomian AKH/ACP and deuterostomian GnRH are orthologs. However, the relationship of CRZ to AKH, ACP and GnRH is less clear. Some studies consider AKH/ACP and CRZ neuropeptides to both be orthologous to the vertebrate GnRH system^1^,^12^,^13^, whilst other studies were inconclusive regarding the evolutionary origins of the CRZ system^2^,^10^.

Informed by analysis of genome sequence data, a candidate neuropeptide (pQILCARAFTYTHTW-NH_2_) in the cephalochordate *Branchiostoma floridae* that activates one of two CRZ-type receptors but neither of two GnRH-type receptors has been reported^14^. It is not known, however, if this predicted mature peptide actually exists in *B. floridae.* Furthermore, a previous report from the same group^15^ showed that the *B. floridae* CRZ-type receptor could also be activated by an insect AKH with equal effectiveness. Thus, it remains to be established whether or not distinct GnRH-type and CRZ-type signalling pathways occur in deuterostomes. Here we have addressed this issue in a non-chordate deuterostomian phylum – the echinoderms.

## Results and Discussion

**Figure 1:**
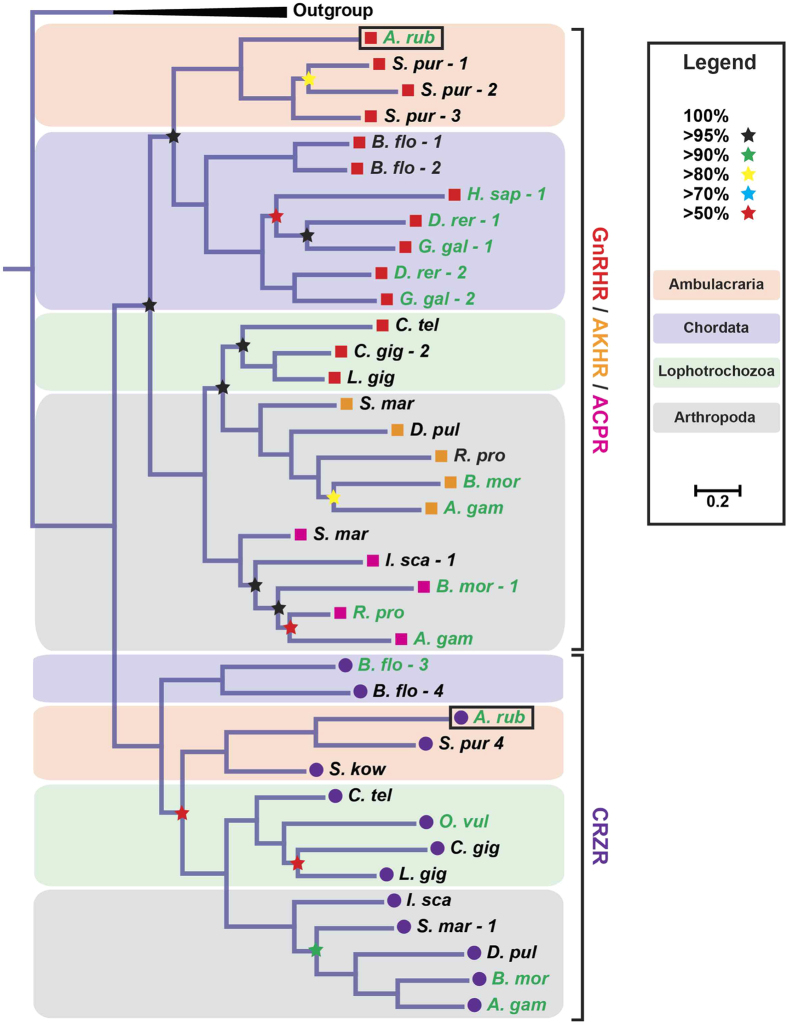
Phylogenetic analysis of GnRH/AKH/ACP/CRZ-type receptors using a Bayesian method reveals two distinct clades–a GnRH/AKH/ACP-type receptor clade and a CRZ-type receptor clade. Single representatives of both clades are present in the starfish *A. rubens* (*A. rub*., black boxes). GnRH-type receptors are labelled using red squares, AKH-type receptors using orange squares, ACP-type receptors using pink squares and CRZ-type receptors using purple circles. Neuropeptide S and CCAP receptors were used as an outgroup (condensed). The stars represent posterior probabilities and the pastel coloured backgrounds represent different groups of animals (see legend). The scale bar indicates amino acid substitutions per site. Species for which receptor-ligand interactions have been experimentally characterized are coloured in green, including the *A. rubens* receptors characterized in this study (boxed). Species names are as follows: A. rub, *Asterias rubens*; S. pur, *Strongylocentrotus purpuratus*; B. flo, *Branchiostoma floridae*; H. sap, *Homo sapiens;* D. rer, *Danio rerio*; G. gal, *Gallus gallus*; C. tel, *Capitella teleta*, C. gig, *Crassostrea gigas*; L. gig, *Lottia gigantea*; S. mar, *Strigamia maritima*; D. pul, *Daphnia pulex*; B. mor, *Bombyx mori*; R. pro, *Rhodnius prolixus*; A. gam, *Anopheles gambiae*; I. sca, *Ixodes scapularis*; S. kow, *Saccoglossus kowalevskii*; O. vul, *Octopus vulgaris*. [accession numbers and references for the receptor sequences are included the legend of Supplementary Figure S3].

Four GnRH/CRZ-type receptors have been identified in the sea urchin *Strongylocentrotus purpuratus* based on analysis of genome sequence data^2^,^14^ but the ligands for these receptors have not been discovered. Here we set out to identify and characterise GnRH/CRZ-type receptors in another echinoderm species–the common European starfish *Asterias rubens*–utilizing neural transcriptome sequence data that has been obtained recently^16^,^17^. BLAST analysis of *A. rubens* neural transcriptome sequence data using an *S. purpuratus* GnRH-type receptor as the query sequence identified two candidate GnRH/CRZ-type receptor transcripts, which we cloned and sequenced as cDNAs (Supplementary Figures S1 and S2). Phylogenetic analysis of the relationships of the two *A. rubens* receptors with GnRH-, AKH-, ACP- and CRZ-type receptors, using Bayesian and maximum-likelihood methods, generated trees with well-supported topologies. With both methods, one receptor grouped with GnRH/AKH/ACP-type receptors and the other grouped with protostomian and *B. floridae* CRZ-type receptors (Fig. 1 and Supplementary Figure S3). Henceforth we will refer to the two receptors as ArGnRHR and ArCRZR, respectively.

**Figure 2:**
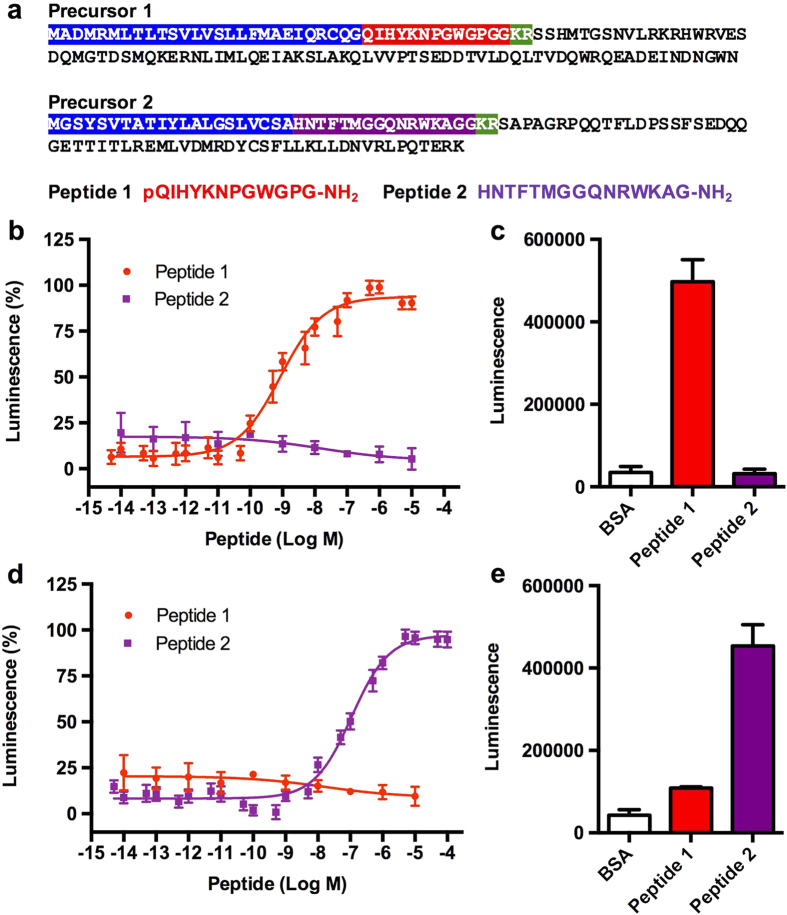
Identification of GnRH-type and Corazonin-type (CRZ)-type signalling systems in the starfish *Asterias rubens.* (**a**) Amino acid sequences of two *A. rubens* GnRH/CRZ-type neuropeptide precursor proteins–precursor 1 and precursor 2. Signal peptides are highlighted in blue, putative neuropeptides (without post-translational modifications) are highlighted in red (peptide 1) or purple (peptide 2) and dibasic cleavage sites are highlighted in green. Peptides 1 and 2 with post-translational N-and C-terminal modifications, determined by mass spectrometry, are shown below the precursor sequences. (**b**) Peptide 1 causes dose-dependent stimulation of a bioluminescence response in CHO-K1 cells stably expressing aequorin and Gα16 and transfected with ArGnRHR; EC_50_ = 6.03 × 10^−10 ^M. Peptide 2 has no effect when tested over the same concentration range as peptide 1, demonstrating the specificity of the activation of ArGnRHR by peptide 1, which is therefore designated as “ArGnRH”. (**c**) Comparison of the total bioluminescent responses of ArGnRHR-expressing cells for 30 seconds after the addition of BSA media (control), peptide 1 (10^−5 ^M) or peptide 2 (10^−5 ^M). (**d**) Peptide 2 causes dose-dependent stimulation of a bioluminescence response in CHO-K1 cells stably expressing aequorin and Gα16 and transfected with ArCRZR; EC_50_ = 1.15 × 10^−7 ^M. Peptide 1 has no effect when tested over a similar concentration range as peptide 2, demonstrating the specificity of the activation of ArCRZR by peptide 2, which is therefore designated as “ArCRZ”. (**e**) Comparison of the total bioluminescent responses of ArCRZR-expressing cells for 30 seconds after the addition of BSA media (control), peptide 1 (10^−5 ^M) or peptide 2 (10^−5 ^M).

Having identified a GnRH-type receptor and a CRZ-type receptor in *A. rubens*, we sought to identify the neuropeptides that act as ligands for these receptors. Analysis of neural transcriptome sequence data has revealed the occurrence of two GnRH/CRZ-type precursors in *A. rubens*^17^ and here we cloned cDNAs encoding these precursors to confirm their sequences (Fig. 2a; Supplementary Figures S4 and S5). Precursor 1 comprises a single copy of the putative GnRH-like peptide pQIHYKNPGWGPG-NH_2_ (peptide 1) whereas precursor 2 comprises a single copy of the putative peptide HNTFTMGGQNRWKAG-NH_2_ (peptide 2). LC-MS-MS analysis of radial nerve cord extracts demonstrated that both of these predicted neuropeptides occur in *A. rubens* (Supplementary Figures S6 and S7).

We hypothesized that the GnRH-like peptide 1 is the ligand for ArGnRHR and peptide 2 is the ligand for ArCRZR and to test this hypothesis the receptors were expressed in a heterologous cellular system. Neither peptide 1 nor peptide 2 elicited any response when tested on cells transfected with an empty vector (not shown) but, consistent with our hypothesis, peptide 1 caused dose-dependent activation of ArGnRHR (EC_50_ = 0.603 nM; Fig. 2b), and peptide 2 caused dose-dependent activation of ArCRZR (EC_50_ = 115 nM; Fig. 2d). Importantly, peptide 1 did not activate ArCRZR (Fig. 2b,c) and likewise peptide 2 did not activate ArGnRHR (Fig. 2d,e), demonstrating the existence of two distinct signalling systems. Neither receptor was activated by other GnRH/CRZ-type peptides (*Drosophila* AKH and CRZ) or by other starfish neuropeptides (NGFFYamide, SALMFamide-1 and SALMFamide-2), providing further evidence of the specificity of peptides 1 and 2 as ligands for ArGnRHR and ArCRZR, respectively (Supplementary Figure S8). Therefore, henceforth we will refer to peptide 1 (pQIHYKNPGWGPG-NH_2_) as ArGnRH and peptide 2 (HNTFTMGGQNRWKAG-NH_2_) as ArCRZ.

ArCRZ is the first ligand for a CRZ-type receptor to be biochemically identified in a deuterostome. Furthermore, precursors of ArCRZ-like peptides can be identified in the sea urchin *S. purpuratus* and the hemichordate *Saccoglossus kowalevskii* (Supplementary Figure S9). Discovery of these ambulacrarian corazonins prompted us to examine the reported precursor of a putative CRZ-type receptor ligand (pQILCARAFTYTHTW-NH_2_) in the cephalochordate *B. floridae*^14^. Analysis of the precursor sequence using the signal peptide prediction tool SignalP 4.1^18^ reveals the presence of a signal peptide cleavage site between the alanine (A) and phenylalanine (F) residues in the middle of the QILCARAFTYTHTW sequence (Supplementary Figure S9). Therefore, the neuropeptide derived from this *B. floridae* precursor protein is predicted to be FTYTHTW-NH_2_. This peptide shares modest sequence similarity with the ambulacrarian corazonins but a feature that unifies deuterostomian CRZ-type precursor genes are two introns that interrupt the protein-coding sequence (Supplementary Figure S9). Furthermore, this feature distinguishes deuterostomian CRZ-type precursor genes from deuterostomian GnRH-type precursor genes, which have a single conserved intron (Supplementary Figure S9).

**Figure 3:**
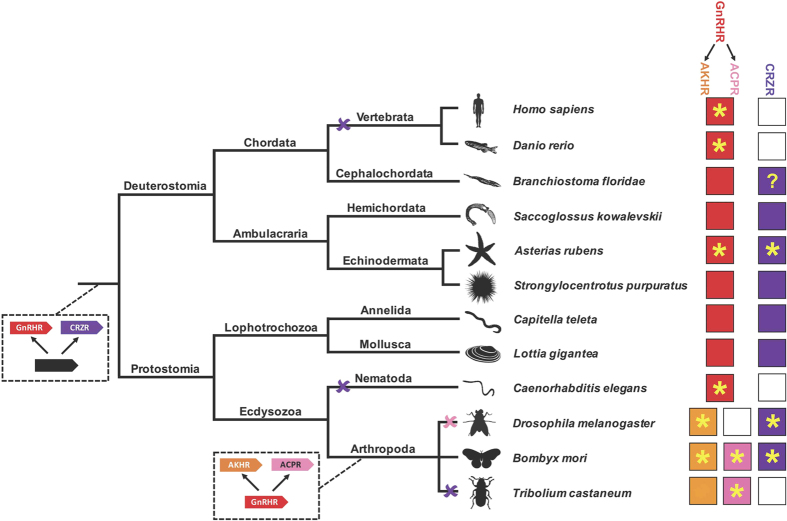
Schematic showing the evolution of GnRH-type and CRZ-type receptors in the Bilateria. GnRH-type receptors (red) and CRZ-type receptors (purple) arose by gene duplication in a common ancestor of the Bilateria. A second gene duplication of a GnRH-type receptor in a common ancestor of the Arthropoda gave rise to AKH-type receptors (orange) and ACP-type receptors (pink). CRZ-type receptors have been lost in multiple lineages (purple crosses), including vertebrates, and the ACP-type receptor has been lost in *Drosophila* (pink cross). The occurrence of each receptor type in species belonging to different phyla is shown on the right (white box denotes loss of a receptor). Species where neuropeptide ligands for receptors have been identified are labelled with a yellow asterisk. Note that, as reported in this paper, the starfish *Asterias rubens* is the first and only deuterostome in which the neuropeptide ligands for a GnRH-type receptor and a CRZ-type receptor have been identified. The “?” in the CRZR box for *Branchiostoma floridae* indicates uncertainty regarding the structure of a candidate ligand, as discussed in the main text of this paper. Images of representative animals from each phylum were created by the authors, with the exception of the sea urchin image, which was obtained from https://openclipart.org/detail/170807/sea-urchin-silhouette.

In conclusion, our discovery of ArCRZ, ArGnRH and their cognate receptors in the starfish *A. rubens*, a deuterostomian invertebrate, indicates that these paralogous signalling systems originated by gene duplication in a common ancestor of the Bilateria (Urbilateria) (Fig. 3). Evidence in support of this conclusion has been obtained previously by Roch *et al*.^14^,^19^ in phylogenetic analyses of GnRH/CRZ-type receptor sequences. Consistent with our findings, trees generated by these authors contain two distinct receptor clades – one comprising GnRH/AKH/ACP-type receptors and another comprising CRZ-type receptors, with receptors from protostomes and deuterostomes in both clades. Furthermore, to enable comparison with the findings of Roch *et al*., in Supplementary Figures S10 and S11 we show neighbour joining and maximum likelihood trees, respectively, that were generated using the same sequences analysed by Roch *et al*.^14^, but with the addition of ArGnRHR and ArCRZR (boxed in red). Consistent with our findings (Fig. 1; Supplementary Figure S3), ArGnRHR is positioned in the GnRHR clade and ArCRZR is positioned in the CRZR clade (Supplementary Figures S10 and S11).

Interestingly, comparison of the sequences of GnRH/AKH/ACP/CRZ neuropeptides and precursor proteins throughout the Bilateria does not reveal any structural characteristics that distinguish CRZ-type from GnRH/AKH/ACP-type neuropeptides/precursors. An explanation for this may be that the gene duplication that gave rise to GnRH/AKH/ACP-type neuropeptides/precursors on the one hand and CRZ-type neuropeptides/precursors on the other occurred just prior to the divergence of protostomes and deuterostomes. Thus, there may have been little or no sequence divergence in the paralogous precursor proteins at the time of the protostome-deuterostome split. An alternative, but less parsimonious, explanation would be that the gene duplications that gave rise to ligands for GnRH-type receptors and CRZ-type receptors occurred independently in both the protostome and deuterostome lineages after the protostome-deuterostome split.

Surveying the occurrence of the GnRH-type and CRZ-type signalling systems throughout the Bilateria reveals that the GnRH-type signalling system appears to have been retained throughout the Bilateria whereas the CRZ-type signalling system has been lost in vertebrates, nematodes and some arthropods (Fig. 3 2,10). In this context, our discovery of both GnRH-type and CRZ-type signalling in an echinoderm is interesting because it has, for the first time, provided a basis for comparison of the physiological roles of these paralogous systems in a deuterostome. Investigation of the actions of GnRH-type neuropeptides has revealed roles in regulation of reproductive processes in chordates^3^,^4^ and in the nematode *C. elegans*^13^. In arthropods, duplication of the GnRH-type signalling system to give rise to the AKH-type and ACP-type signalling systems complicates the picture. AKH regulates lipid-mobilisation in insects^5^ but the physiological roles of the more recently discovered ACP have yet to be well characterised^11^. In this context, it will be interesting to determine the physiological roles of a GnRH-type neuropeptide in an echinoderm, as this will serve as a “bridge” between vertebrates and protostomes in our knowledge and understanding of the evolution of GnRH function. Likewise, whilst much is now known about the physiological roles of CRZ-type signalling in arthropods^6^,^7^,^8^, nothing is known about the physiological roles of this signalling system in deuterostomes. Our discovery of ArCRZ and ArCRZR provides a unique opportunity to address this issue.

## Materials and Methods

### Identification and cloning of GnRH/CRZ-type receptors and neuropeptide precursors in *A. rubens*

Two putative GnRH-type neuropeptide precursors have been identified recently in *A. rubens*^17^ and two candidate GnRH/CRZ-type receptors for peptides derived from these precursors were identified by BLAST analysis of *A. rubens* radial nerve cord transcriptome sequence data using a *S. purpuratus* GnRH-type receptor as the query. Then cDNAs encoding these precursor proteins and receptors were cloned and sequenced, using specific primers (see Supplementary Figures S1 and S2) designed using Primer3 online tool (http://primer3.ut.ee).

### Phylogenetic Analysis

Phylogenetic analysis of the relationship of *A. rubens* GnRH/CRZ-type receptors with GnRH-, AKH-, ACP- and CRZ-type receptors from other species was accomplished using Bayesian and maximum-likelihood methods^11^. The sequences were aligned using the MAFFT v7.017 plugin in Geneious 8.0.5 (slow iterative, maximum 1000 iterations, BLOSUM30)^20^. The alignment was then trimmed using BMGE with the following options: BLOSUM30, max –h = 1, −b = 1^21^. The maximum-likelihood tree was produced using PhyML 3.0 (LG substitution model, 1000 Bootstrap)^22^. The Bayesian tree was produced using Mr Bayes version 3.2.1 (WAG model, +I + G + F, 2 runs, 1000000 trees; burn-in 10%). The consensus tree was created in Geneious 8.0.5.

### Mass spectrometry

Two different methods were used to prepare extracts of radial nerve cords from *A. rubens*, with radial nerve cords from two animals used for each method. For an acetic acid based extraction, nerve cords were dissected and transferred to a micro-centrifuge tube containing 3% acetic acid (in ddH_2_O). The tube was incubated in a boiling water bath for 10 minutes. The nerve cords were then sonicated and homogenized to lyse cells. The extract was centrifuged and the supernatant transferred to a glass vial. For a second method, nerve cords were dissected and transferred to a 90% methanol and 9% acetic acid solution. The nerve cords were sonicated and homogenized to lyse cells. The extract was centrifuged and the supernatant transferred to a glass vial. Finally, the solvent was bubbled-off using nitrogen gas. The extract was analysed by means of nanoflow liquid chromatography with electrospray ionisation quadrupole time-of-flight tandem mass spectrometry (nanoLC-ESI-MS/MS) using a nanoAcquity UPLC system coupled to a Synapt G2 HDMS mass spectrometer (Waters Corporation, Milford, MA, USA) and MassLynx v4.1 SCN 908 software (Waters Corporation, Milford, MA, USA). All MS/MS samples were analyzed using Mascot (Matrix Science, London, UK; version 2.5.0) and Scaffold (version Scaffold_4.2.1, Proteome Software Inc., Portland, OR) was used to validate MS/MS based peptide and protein identifications. [For a more detailed account of the mass spectrometry methods employed, see Supplementary Figure S6.]

### Functional characterization of ArGnRHR and ArCRZR

ArGnRHR and ArCRZR were cloned as described previously^23^ and the positions of the primers used are indicated in Supplementary Figures S1 and S2. Next, the ORF of ArGnRHR was amplified using the oligos 5′-aagcttCACCATGGCGACTACATC-3′ and 5′-ctcgagTTATACACATTTCTCAG-3′ and subcloned into the eukaryotic expression vector pcDNA 3.1+ (Invitrogen) that was cut with *Hind*III and *Xho*I; The ORF of ArCRZR was amplified using the oligos 5′- gatatcCACCATGAGTGTTCAAT-3′ and 5′-tctagaTCAGGTTGTTGTTGTGA-3′ and subcloned into pcDNA 3.1+ that was cut with *EcoR*V and *Xba*I. A partial Kozak translation initiation sequence (CACC) was also introduced in the upstream primer. Chinese hamster ovary (CHO)-K1 cells stably overexpressing the human Gα16 protein and mitochondrial targeted apo-aequorin were used as a heterologous expression system to functionally characterize the receptors. Cells were cultured, transfected and a bioluminescence assay performed as described previously^23^. The *A. rubens* neuropeptides pQIHYKNPGWGPG-NH_2_ and HNTFTMGGQNRWKAG-NH_2_ were custom synthesized by PPR Ltd (Fareham, UK) and were tested as candidate ligands for ArGnRHR and ArCRZR at concentrations ranging from 10^−4 ^M to 10^−14 ^M. Ca^2+^ responses were normalized to the total Ca^2+^ response monitored after addition of Triton X-100 (0.1%). Dose–response data were determined as a % of the highest response (100% activation). EC_50_ values were calculated from dose–response curves based on at least three independent transfections (Prism 6.0). Other GnRH/CRZ-type neuropeptides (*Drosophila* AKH and *Drosophila* AKH) and other starfish neuropeptides (NGFFYamide, SALMFamide-1 and SALMFamide-2^16^,^24^) were also tested (at 10 μM) to assess the specificity of receptor activation.

## Additional Information

**How to cite this article**: Tian, S. *et al*. Urbilaterian origin of paralogous GnRH and corazonin neuropeptide signalling pathways. *Sci. Rep.* **6**, 28788; doi: 10.1038/srep28788 (2016).

## Supplementary Material

Supplementary Information
