# Supplementary material for: Urbilaterian origin of paralogous GnRH and corazonin neuropeptide signalling pathways
Source: Sci Rep. 2016 Jun 28;6:28788. doi: 10.1038/srep28788 (PMC4923880; doi:10.1038/srep28788)
Supplement: Supplementary Information [file srep28788-s1.pdf]

Urbilaterian origin of paralogous GnRH and corazonin neuropeptide signalling pathways

Shi Tian, Meet Zandawala, Isabel Beets, Esra Baytemur,

Susan E. Slade, James H. Scrivens, Maurice R. Elphick

SUPPLEMENTARY INFORMATION

```
1       cagtcttcatcagagtcggcaaccataccgcatgggtcggacggatacccaggatatctcagttgaactgcggttagaaa
81      ccaagaagattcctacacagtcagtctagcctccgactccaacagttgtttcgtgaatacttctgcattgactccccgtt
161     gcaggtaaaagcaattttggaatgcgaatctggattgaagttcgctgatattacctacgtagcttaccaatattgcacca
241     gttggatagctgatgaaacgcaacgcaatcctccatcaagatatgagaggtgagtaacctcaggggaatgcacttcgatg
321     ctaacagagagggactcaaggacgtcatatcgcccgccctcgcacggtcagaatcaaccagtctacaaaaaaccggggct
401     cactgtagtaacgtgtcctcggctcagatcacacacgcagtgaacatctgatgatgtgtgataaagtgaacactcttaga
481     gtaagatcatcagttgcttcttgttactggttgaacttccagaaggaacactcgaactttgaacatcttatgatgtgtat
561     cgaacaatcttagagttgatctctagttacttcttgttacgcttttgaacttcccgaagtaacactggcgttgtgaacat
641     ctgacgatgtgtatggaaccctcttagaatagatcgccagttgctgcttgtaacgttttcaactccctggaggaactatt
721     catttcttgctttggttcgcaatctgtaaacaccatggcgactacatcagtcaatccaactttgataacttacatttatg
                                           M  A  T  T  S  V  N  P  T  L  I  T  Y  I  Y  15
801     agacagtggaagtgaatgatacctactccaacttcaccaaagcgggcccgacgtctgatggcaatggaaccgactatcat
        E  T  V  E  V  N  D  T  Y  S  N  F  T  K  A  G  P  T  S  D  G  N  G  T  D  Y  H  42
881     tcaggtattagcatttatgatgattacttaatacgactgattctctacgtgattatattcgtagtctccacaataggaaa
         S  G  I  S  I  Y  D  D  Y  L  I  R  L  I  L  Y  V  I  I  F  V  V  S  T  I  G  N 69
961     cactgcggtattgtgtagtctcatcaaaggacgcaggaggaagtctcgggtgaatcttctcatcatgcacttgactgtag
          T  A  V  L  C  S  L  I  K  G  R  R  R  K  S  R  V  N  L  L  I  M  H  L  T  V  95
1041    cagatcttatgataacgttcttcaatatcccaacatacttcatctggctcattacataccagtggtacggaggggatatc
        A  D  L  M  I  T  F  F  N  I  P  T  Y  F  I  W  L  I  T  Y  Q  W  Y  G  G  D  I  122
1121    atgtgtaggtccgtcatgtacattgccatggtagggacatacgcgtcgccattcattctcatcgtcataagtctagaccg
         M  C  R  S  V  M  Y  I  A  M  V  G  T  Y  A  S  P  F  I  L  I  V  I  S  L  D  R 149
1201    gtttgcctctattgtattcccacttagcgtccggcaggcagatatgaggtgtaagattatgctacgggttgcgtgggcag
          F  A  S  I  V  F  P  L  S  V  R  Q  A  D  M  R  C  K  I  M  L  R  V  A  W  A  175
1281    cgtgtatcatcgcaagcattccccagcttcttatccatcaagtcatgtcaccgaagagtgacccagacttcactcagtgt
        A  C  I  I  A  S  I  P  Q  L  L  I  H  Q  V  M  S  P  K  S  D  P  D  F  T  Q  C  202
1361    gttgactatggattcagaaagaactttcctgtattctggaacttataccattggttcgtcatggctgccacgtactttat
         V  D  Y  G  F  R  K  N  F  P  V  F  W  N  L  Y  H  W  F  V  M  A  A  T  Y  F  I 229
1441    acctttgacgttgattattggctgctatacttccattgtcatcaaaatatttggcaatagcaccatccgcagctattccg
          P  L  T  L  I  I  G  C  Y  T  S  I  V  I  K  I  F  G  N  S  T  I  R  S  Y  S  255
1521    gtaacaatggcaaccgaatgacgcttcgtcgctccggtgtcgacactctccctaaggctcgcgtgagggcgcttaagatg
        G  N  N  G  N  R  M  T  L  R  R  S  G  V  D  T  L  P  K  A  R  V  R  A  L  K  M  282
1601    actggtgcaatcgtcacagccttcatagtatgctggactccaacgtgcattgagggcactatcacccacgcaaaccctgc
         T  G  A  I  V  T  A  F  I  V  C  W  T  P  T  C  I  E  G  T  I  T  H  A  N  P  A 309
1681    tctgggagaagcaaccccgatatggttgaatcatataatgctagcatttggcttctctaatgtttgtatcgacccaatag
          L  G  E  A  T  P  I  W  L  N  H  I  M  L  A  F  G  F  S  N  V  C  I  D  P  I  335
1761    tctatgggatgttcaccgtggactttaggcggcatttcccagggtgtttcgactgctggggcgggcggaatatcctgcgg
        V  Y  G  M  F  T  V  D  F  R  R  H  F  P  G  C  F  D  C  W  G  G  R  N  I  L  R  362
1841    ggtcgacgcagtggaaggtccaccacgtcgtctaacaactatccacccgtcacctatgcgtcttccactcgatgtggtac
         G  R  R  S  G  R  S  T  T  S  S  N  N  Y  P  P  V  T  Y  A  S  S  T  R  C  G  T 389
1921    cactacaggcgtgtcagggaactatcatgtgatggaattggatagtcgccatgggacagctgagaaatgtgtataaagca
          T  T  G  V  S  G  N  Y  H  V  M  E  L  D  S  R  H  G  T  A  E  K  C  V  *     413
2001    gtgctttatttgtgtgatgtcaagagcagagatcatgccacaaaagaagatctaatggttacgaccctagggagaatgtc
2081    tttatacattactatcatcttaataagaaacaatctcgacaaatctccaacaagactctagcataaaacaaaagcatcca
2161    gtcaatgcattcattctgagtcaaacactccgtatacacacatgattattctgtggataaatgaagataaagcaagaata
2241    attttcagtattctcctgaagacgagcagaatgtactgttcgaaacgtcgagac
```

Supplementary Figure S1. ***Asterias rubens* GnRH-type receptor (ArGnRHR).** The transcript cDNA sequence (lowercase; numbering on the left) and the deduced amino acid sequence of the protein coding region (uppercase; numbering on the right) are shown. Within the amino acid sequence, the predicted transmembrane regions are highlighted in black and potential N-linked glycosylation sites are in yellow. The asterisk denotes the stop codon. The transcript sequence encoding ArGnRHR (contig 1119703) was first identified by BLAST analysis of *A. rubens* radial nerve transcriptome sequence data. A cDNA corresponding to the protein-coding region the ArGNRHR transcript was then was amplified by PCR from *A. rubens* radial nerve cord cDNA using the primers indicated in the boxes. The cDNA was cloned in the vector pBluescript and T3 and T7 primers were used for sequencing. The cDNA sequence was found to be identical to the protein coding region of contig 1119703. This sequence has been submitted to the GenBank database under accession number KU888680.

```
1      atcagttgggatccgattaacaggattctaagcaaaggttttcgacattcttgctgacaaatataaaacttgtttaagct
81     tttaaaaagtggtcaaagctttttggataaaatccaagatgagtgttcaatacacatcagatgagagtgaattcgcgtta
                                                                M  S  V  Q  Y  T  S  D  E  S  E  F  A  L   14
161    tgtgacttactatcttcagctaccgaatgcgatgagaacactactatcgccttacctgaattcacgccattcacggcgtt
        C  D  L  L  S  S  A  T  E  C  D  E  N  T  T  I  A  L  P  E  F  T  P  F  T  A  F  41
241    caaggtgggcgtcttggctctcatgatagtattttccaccgttggaaactgcattgccattgccgtcacctgcaagatcc
         K  V  G  V  L  A  L  M  I  V  F  S  T  V  G  N  C  I  A  I  A  V  T  C  K  I   67
321    gtggtcgccggaaatccacagtgactacgctgatcctaaacctggcagtcagtgacctcatcgtgacgtactgccacatg
       R  G  R  R  K  S  T  V  T  T  L  I  L  N  L  A  V  S  D  L  I  V  T  Y  C  H  M   94
401    ctggtccatatgatatggtactccaccgacgcatggctaggtggggaagctctgtgtaaaatcgctaagttcttctctaa
        L  V  H  M  I  W  Y  S  T  D  A  W  L  G  G  E  A  L  C  K  I  A  K  F  F  S  N  121
481    ctttggcttatttgcttcatccttcatcactgtggatgttgggttggacaggtgcctggcagtacttcgcccgttggggc
         F  G  L  F  A  S  S  F  I  T  V  D  V  G  L  D  R  C  L  A  V  L  R  P  L  G   147
561    accgtcagcgaccgttccatattaagatgatgattataacctcgtacatggttgcctttctcttcagtattccacagctt
       H  R  Q  R  P  F  H  I  K  M  M  I  I  T  S  Y  M  V  A  F  L  F  S  I  P  Q  L   174
641    atcgttttccgtttagagcactggccgttcgatcctgaaatcgacttctggcagtgtgtgacaaacgttggcatccctaa
        I  V  F  R  L  E  H  W  P  F  D  P  E  I  D  F  W  Q  C  V  T  N  V  G  I  P  N  201
721    tgtttatatcgccgtgtatactacactggtagtcttggcccagttcgtggccccattgggcatcatgatggttgcgtatg
         V  Y  I  A  V  Y  T  T  L  V  V  L  A  Q  F  V  A  P  L  G  I  M  M  V  A  Y   227
801    ggctcatcttcatgaaagtgcgacaaaagatcgtcatgaaagaaaccagcgagaatctgtcagagatgcgtcaagctcgc
       G  L  I  F  M  K  V  R  Q  K  I  V  M  K  E  T  S  E  N  L  S  E  M  R  Q  A  R   254
881    tccaagctgttccttcgagcccagagacgtacagtgcgcatggcagtcgccatttttatcgtgtttgccatcaactggtt
        S  K  L  F  L  R  A  Q  R  R  T  V  R  M  A  V  A  I  F  I  V  F  A  I  N  W  L  281
961    accctacgccatctttggtctttggtatgtctggttcccaggccaaacttataacatgtacgtgtttgaggtggctttca
         P  Y  A  I  F  G  L  W  Y  V  W  F  P  G  Q  T  Y  N  M  Y  V  F  E  V  A  F   307
1041   tgtttggtctgtctaactcctgcttcaacccactcatctatggagcttgtaacgtgcggtattgtcacaagatctgcgcc
       M  F  G  L  S  N  S  C  F  N  P  L  I  Y  G  A  C  N  V  R  Y  C  H  K  I  C  A   334
1121   tgttttgggatccgttccaagccaggcatggacacgagccactcagaccgctccaacaagacgactatgtactccgtgcg
        C  F  G  I  R  S  K  P  G  M  D  T  S  H  S  D  R  S  N  K  T  T  M  Y  S  V  R  361
1201   ttggcagtcatctaaaggaggtaacaacacacacaagaataataataaggacaatagctggactaaaggcacagcagctg
         W  Q  S  S  K  G  G  N  N  T  H  K  N  N  N  K  D  N  S  W  T  K  G  T  A  A   387
1281   taccacctgagaggcaacagatgatcacaacaacaacctgaagtattaaatcactaaatgacgtccagtattgttgatgg
       V  P  P  E  R  Q  Q  M  I  T  T  T  T  *                                          400
1361   ttgttcatgttaagttgttacaacagatcatttaatccgcattgtaaatagagcggtttccatagtttaaaacaaaggca
1441   ttgaatgacgggtctctaggtgcttcagcaaaaaaaaaaaaaaaaaa
```

Supplementary Figure S2. ***Asterias rubens*** **corazonin-type receptor (ArCRZR)**. The transcript cDNA sequence (lowercase; numbering on the left) and the deduced amino acid sequence of the protein coding region (uppercase; numbering on the right) are shown. Within the amino acid sequence, the predicted transmembrane regions are highlighted in black and a potential N-linked glycosylation site is in yellow. The asterisk denotes the stop codon. The transcript sequence encoding ArCRZR (contig 1109374) was first identified by BLAST analysis of *A. rubens* radial nerve transcriptome sequence data. A cDNA was amplified by PCR from *A. rubens* radial nerve cord cDNA using the primers indicated in the boxes. The cDNA was cloned in the vector pBluescript and T3 and T7 primers were used for sequencing. The bases highlighted in red are different to those in contig 1109374 but all of these are synonymous substitutions. This sequence has been submitted to the GenBank database under accession number KU888681.

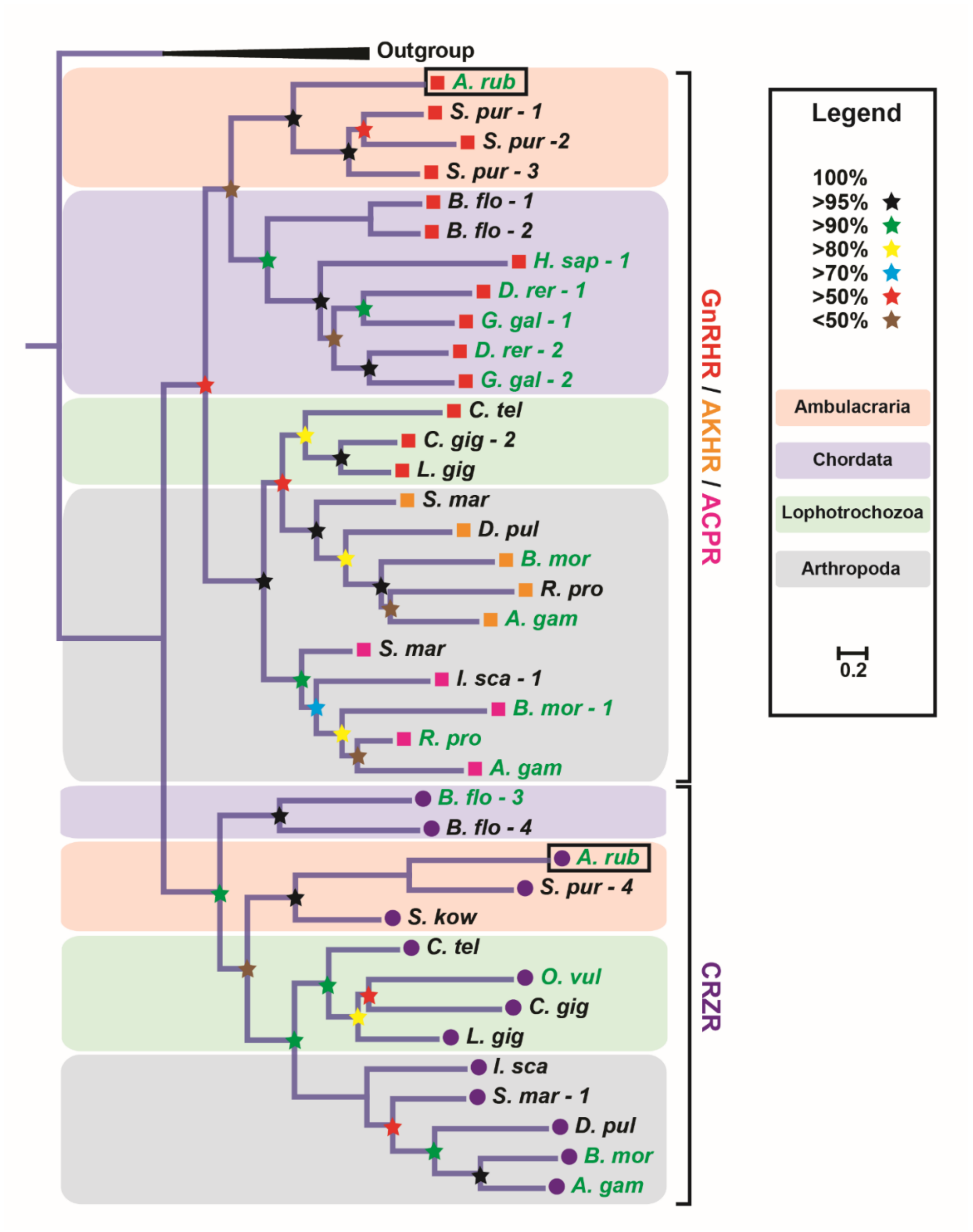

Supplementary Figure S3. Phylogenetic tree (maximum likelihood) of GnRH/AKH/ACP-type and CRZ-type receptors. GnRH-type receptors are labelled using red squares, AKH-type receptors using orange squares, ACP-type receptors using pink squares and CRZ-type receptors using purple circles. Neuropeptide S and CCAP receptors were used as an outgroup (condensed). The stars represent bootstrap support and the shaded backgrounds represent different animal groups (see legend). The scale bar indicates amino acid substitutions per site. Species for which receptor-ligand interactions have been experimentally determined are coloured in green and the *A. rubens* receptors characterized in this study are boxed. Note that GnRH-, AKH- and ACP-type receptors on the one hand and CRZ receptors on the other are grouped in two distinct clades. Species names are as follows: A. rub, *Asterias rubens*; S. pur, *Strongylocentrotus purpuratus*; B. flo, *Branchiostoma floridae*; H. sap, *Homo sapiens;* D. rer, *Danio rerio*; G. gal, *Gallus gallus*; C. tel, *Capitella teleta*, C. gig, *Crassostrea gigas*; L. gig, *Lottia gigantea*; S. mar, *Strigamia maritima*; D. pul, *Daphnia pulex*; B. mor, *Bombyx mori*; R. pro, *Rhodnius prolixus*; A. gam, *Anopheles gambiae*; I. sca, *Ixodes*

scapularis; S. kow, *Saccoglossus kowalevskii*; O. vul, *Octopus vulgaris*. The accession numbers and references for the receptor sequences are as follows:

GnRH/AKH/ACP-type receptors –

GnRH receptors:

A. rub, KU888680; S. pur-1, NP_001116990.1, (Sodergren, et al. 2006) ; S. pur-2, NP_001116992, (Sodergren, et al. 2006); S. pur-3, NP_001116991, (Sodergren, et al. 2006) ; B. flo-1, ACC68665.1, (Tello and Sherwood 2009); B. flo-2, ACC68666.1, (Tello and Sherwood 2009) ; H. sap-1, NP_000397.1, (Kakar, et al. 1992) ; D. rer-1, NP_001138452, (Tello, et al. 2008) ; G. gal-1, NP_989984, (Sun, et al. 2001) ; D. rer-2, NP_001138451, (Tello, et al. 2008) ; G. gal-2, NP_001012627.1, (Shimizu and Bedecarrats 2006) ; C. tel, ELT93721.1, (Simakov, et al. 2013) ; C. gig-2, from WGS, AFTI01018035.1, (Zhang, et al. 2012) ; L. gig, ESP05621, (Simakov, et al. 2013) ;

AKH receptors:

S. mar, AFFK01020326.1, (Chipman, et al. 2014); D. pul, ACD75498, (Colbourne, et al. 2011) ; B. mor, NP_001037049.1, (Hansen, et al. 2010) ; R. pro, KF534791, (Zandawala, Hamoudi, et al. 2015) ; A. gam, ABD60146.1, (Kaufmann and Brown 2006) ;

ACP receptor:

S. mar, AFFK01021969.1 and AFFK01021968.1, (Chipman, et al. 2014); I. sca-1, from WGS, ABJB010361713.1 and ABJB010787461.1, (Ayllon, et al. 2015) ; B. mor-1, NP_001127726.1, (Hansen, et al. 2010) ; R. pro, AKO62856.1,(Zandawala, Haddad, et al. 2015) ; A. gam, ABX52399.1, (Hansen, et al. 2010);

CRZ-type receptors –

B. flo-3, ACC68668.1, (Tello and Sherwood 2009) ; B. flo-4, ACN79527.1, (Tello and Sherwood 2009) ; A rub, KU888681; S. pur-4, XP_011680711.1, (Sodergren, et al. 2006) ; S. kow, XP_006819806.1, (Simakov, et al. 2015) ; C. tel, ELT93721.1, (Simakov, et al. 2013) ; O. vul, Q2V2K5, (Kanda, et al. 2006) ; C. gig, from WGS, AFTI01018035.1, (Zhang, et al. 2012) ; L. gig, ESP05621, (Simakov, et al. 2013) ; I. sca, from WGS, ABJB010847221.1 and ABJB01088645.1, (Ayllon, et al. 2015) ; S. mar-1, from WGS, AFFK01019957.1, (Chipman, et al. 2014) ; D. pul, EFX87464.1, (Colbourne, et al. 2011) ; B. mor, NP_001127719.1, (Hansen, et al. 2010) ; A. gam, AAQ67361.1, (Belmont, et al. 2006) .

References

Ayllon N, Villar M, Galindo RC, Kocan KM, Sima R, Lopez JA, Vazquez J, Alberdi P, Cabezas-Cruz A, Kopacek P, et al. 2015. Systems biology of tissue-specific response to Anaplasma phagocytophilum reveals differentiated apoptosis in the tick vector Ixodes scapularis. PLoS Genet 11:e1005120.

Belmont M, Cazzamali G, Williamson M, Hauser F, Grimmelikhuijzen CJ. 2006. Identification of four evolutionarily related G protein-coupled receptors from the malaria mosquito Anopheles gambiae. Biochem Biophys Res Commun 344:160-165.

Chipman AD, Ferrier DE, Brena C, Qu J, Hughes DS, Schroder R, Torres-Oliva M, Znassi N, Jiang H, Almeida FC, et al. 2014. The first myriapod genome sequence reveals conservative arthropod gene content and genome organisation in the centipede Strigamia maritima. PLoS Biol 12:e1002005.

Colbourne JK, Pfrender ME, Gilbert D, Thomas WK, Tucker A, Oakley TH, Tokishita S, Aerts A, Arnold GJ, Basu MK, et al. 2011. The ecoresponsive genome of Daphnia pulex. Science 331:555-561.

Hansen KK, Stafflinger E, Schneider M, Hauser F, Cazzamali G, Williamson M, Kollmann M, Schachtner J, Grimmelikhuijzen CJ. 2010. Discovery of a novel insect neuropeptide signaling system closely related to the insect adipokinetic hormone and corazonin hormonal systems. J Biol Chem 285:10736-10747.
Kakar SS, Musgrove LC, Devor DC, Sellers JC, Neill JD. 1992. Cloning, sequencing, and expression of human gonadotropin releasing hormone (GnRH) receptor. Biochem Biophys Res Commun 189:289-295.
Kanda A, Takahashi T, Satake H, Minakata H. 2006. Molecular and functional characterization of a novel gonadotropin-releasing-hormone receptor isolated from the common octopus (Octopus vulgaris). Biochem J 395:125-135.
Kaufmann C, Brown MR. 2006. Adipokinetic hormones in the African malaria mosquito, Anopheles gambiae: identification and expression of genes for two peptides and a putative receptor. Insect Biochem Mol Biol 36:466-481.
Shimizu M, Bedecarrats GY. 2006. Identification of a novel pituitary-specific chicken gonadotropin-releasing hormone receptor and its splice variants. Biol Reprod 75:800-808.
Simakov O, Kawashima T, Marletaz F, Jenkins J, Koyanagi R, Mitros T, Hisata K, Bredeson J, Shoguchi E, Gyoja F, et al. 2015. Hemichordate genomes and deuterostome origins. Nature 527:459-465.
Simakov O, Marletaz F, Cho SJ, Edsinger-Gonzales E, Havlak P, Hellsten U, Kuo DH, Larsson T, Lv J, Arendt D, et al. 2013. Insights into bilaterian evolution from three spiralian genomes. Nature 493:526-531.
Sodergren E, Weinstock GM, Davidson EH, Cameron RA, Gibbs RA, Angerer RC, Angerer LM, Arnone MI, Burgess DR, Burke RD, et al. 2006. The genome of the sea urchin Strongylocentrotus purpuratus. Science 314:941-952.
Sun YM, Flanagan CA, Illing N, Ott TR, Sellar R, Fromme BJ, Hapgood J, Sharp P, Sealfon SC, Millar RP. 2001. A chicken gonadotropin-releasing hormone receptor that confers agonist activity to mammalian antagonists. Identification of D-Lys(6) in the ligand and extracellular loop two of the receptor as determinants. J Biol Chem 276:7754-7761.
Tello JA, Sherwood NM. 2009. Amphioxus: beginning of vertebrate and end of invertebrate type GnRH receptor lineage. Endocrinology 150:2847-2856.
Tello JA, Wu S, Rivier JE, Sherwood NM. 2008. Four functional GnRH receptors in zebrafish: analysis of structure, signaling, synteny and phylogeny. Integr Comp Biol 48:570-587.
Zandawala M, Haddad AS, Hamoudi Z, Orchard I. 2015. Identification and characterization of the adipokinetic hormone/corazonin-related peptide signaling system in Rhodnius prolixus. Febs j 282:3603-3617.
Zandawala M, Hamoudi Z, Lange AB, Orchard I. 2015. Adipokinetic hormone signalling system in the Chagas disease vector, Rhodnius prolixus. Insect Mol Biol 24:264-276.
Zhang G, Fang X, Guo X, Li L, Luo R, Xu F, Yang P, Zhang L, Wang X, Qi H, et al. 2012. The oyster genome reveals stress adaptation and complexity of shell formation. Nature 490:49-54.

```
1    aaaaactgatttgcttaagctggaagtaacaaaatattgatcgattgtcttgaaagatga
61   acctgtcccagttcattcttcggagacattactctgtattaagatcaatccacgtgtgat
121  gaaggttacggatacgctgagacgtcaattgatgtcactggagtcgacaaatgcgtgcac
181  gccgattagttgctagacttttagccaatggcgcactgttgctcagctcacgtgataat
241  accgtcaactgtccccttttcactcatttcggttccttgttttaaagcatcagacgtgac
301  ctcttgagattgatcaaaaagagctttatatgttcagtgtcaactcattatttcagcaga
361  taaagaccgtcagagtgatttttcacacagtatttacgtttatagtcatcgttaatagtt
421  cacgtcacgaccactgggctacattaacacccatctatagatccgcgtaacactcccaag
481  cccagctatctgttataccggtaaccattaggtggagattgcctgccatacatttatgcg
541  cacgcgacacagcgtgcaagtctcagttcgttctcagttagcaaccaagaaatagtgtaa
601  gcgcttcataaggaaaactgtaagagaagaacacaggagagtcactggagttaagaagcc
661  caagtcaccttataaggtaattttgtacagatggccgatatgaggatgttaacactcact
                                    M  A  D  M  R  M  L  T  L  T   10
721  agcgtattagtctctactcttcatggcagaaattcaaagatgccaagggcagatacat
      S  V  L  V  S  L  L  F  M  A  E  I  Q  R  C  Q  G  Q  I  H   30
781  tacaagaatcctggatggggacctggtggtaaaaggagttcacacatgactggtagcaat
      Y  K  N  P  G  W  G  P  G  G  K  R  S  S  H  M  T  G  S  N   50
841  gtattaaggaaacggcattggcgcgtggaatctgatcagatgggtacagacagcatgcag
      V  L  R  K  R  H  W  R  V  E  S  D  Q  M  G  T  D  S  M  Q   70
901  aaagaacgaaacttgatcatgcttcaagaaattgcaaaatctttggcaaagcaactggta
      K  E  R  N  L  I  M  L  Q  E  I  A  K  S  L  A  K  Q  L  V   90
961  gtaccaacgagtgaggacgacacagtcctggaccaattaacggtcgaccaatggcggcag
      V  P  T  S  E  D  D  T  V  L  D  Q  L  T  V  D  Q  W  R  Q   110
1021 gaagcagacgagataaatgacaacggttggaattaagcgggaaaagctctgaaatttgac
      E  A  D  E  I  N  D  N  G  W  N  *                           121
1081 aacaattatttagaatcaggaagaactgaacaacttgatacaggttcatatgtgtttgta
1141 ttgctttcttttt
```

Supplementary Figure S4. ***Asterias rubens*** **GnRH-type precursor (ArGnRHP).** The transcript cDNA sequence (lowercase; numbering on the left) and the deduced amino acid sequence (uppercase; numbering on the right) are shown. Within the amino acid sequence, the predicted signal peptide is highlighted in blue, the mature peptide sequence is highlighted in red, a putative dibasic cleavage site (KR) is highlighted in green and a GnRH-associated peptide is highlighted in grey. The asterisk denotes the stop codon. The transcript sequence encoding ArGnRHP (contig 1100532) was first identified by BLAST analysis of *A. rubens* radial nerve transcriptome sequence data (see ArGnRH1P in Semmens et al., 2016). A cDNA was amplified by PCR from *A. rubens* radial nerve cord cDNA using primers corresponding to the sequences highlighted in yellow. The cDNA was cloned in the vector pBluescript and T3 and T7 primers were used for sequencing. The cDNA sequence was found to be identical to the corresponding region of contig 1100532. This sequence has been deposited in GenBank database under accession number KT601712.

```
1                                                               g
2     gactccggacatcatctgtagttgatctatcaatcgcgttcgaagacgatggggagttac
                                                          M  G  S  Y   4
62    tcggttacggccaccatatacctagccctagttttaggttctttagtatgtagcgcccac
       S  V  T  A  T  I  Y  L  A  L  V  L  G  S  L  V  C  S  A  H 24
122   aatacgttcaccatgggtggacagaacaggtggaaagcaggggaaagagatcggctccc
       N  T  F  T  M  G  G  Q  N  R  W  K  A  G  G  K  R  S  A  P 44
182   gcagggcgccctcaacaaactttcttggacccatcaagcttcagtgaagatcagcaagga
       A  G  R  P  Q  Q  T  F  L  D  P  S  S  F  S  E  D  Q  Q  G 64
242   gaaacgacaattacgctacgggagatgctggtcgacatgagagactactgcagtttcctc
       E  T  T  I  T  L  R  E  M  L  V  D  M  R  D  Y  C  S  F  L 84
302   ttgaagctacttgacaacgttcggctgcctcaaaccgaaagaaaatgacacttctaagat
       L  K  L  L  D  N  V  R  L  P  Q  T  E  R  K  *             99
362   gaaatcagacgccagactactataccacgtctatctttgtctatcttgcgaaatggagga
422   ggctgtgcccagacatgaattgcaggaccttattaccagggagaccgtgatgggcagttc
482   cgattctctactttagagtagtgacgtcattccgcaggataaaaaatgatggatgaaaat
542   cgactgagagggaccacccttgtgcttgggggtttactctccctcaacacagcgactat
602   gaaattaaacgagacaactgaaaagtgaaaacgacaaacttgtatattttcagaatagta
662   catcagccccttttcgattgtgttttggggtgtgtacgcgggcgcctttattctccaacg
722   tataaaaacctcctttgaccacacagctactaacaattgacattgctatcattcaattat
782   tcccgccgatattttcgacggtcctttttttctttcttcaaaacacccgtaatttttttt
842   gaacacgtggggattgaagcgtttttgagaaaacagttttctaaaaataaaccgatacga
902   ttaatttcccagccatttgacgtttaaaaacaagaaggccctaccatactgcgtacattt
962   ttgatgttccctgttcagttcatcctttaaaattttacaaattactctatgaaaaccta
1022  caaaccactagttagattctaaaagcttattgcttttggaattctagtcccataagaaag
1082  caaaatattcgtgttaagcatacaaatgtgcttaggcgtaagcgtatttttacaggttaa
1142  caagacaattgagcggccagctattgcagccaaggtttaccatcaataagcaccggaagg
1202  ttagcatgctttaagagatgaaattccccttaataataataataattattacgagtccaa
1262  tataaggtgtaaaccacgttcagcaaaggtcaatgt
```

Supplementary Figure S5. ***Asterias rubens*** **corazonin-type precursor (ArCRZP).** The transcript cDNA sequence (lowercase; numbering on the left) and the deduced amino acid sequence (uppercase; numbering on the right) are shown. Within the amino acid sequence, the predicted signal peptide is highlighted in blue, the mature peptide sequence is highlighted in red, a putative dibasic cleavage site (KR) is highlighted in green and a corazonin-associated peptide (CAP) is highlighted in grey. The asterisk denotes the stop codon. The transcript sequence encoding ArCRZP (contig 1104992) was first identified by BLAST analysis of *A. rubens* radial nerve transcriptome sequence data (see ArGnRH2P in Semmens et al., 2016). A cDNA was amplified by PCR from *A. rubens* radial nerve cord cDNA using primers corresponding to the sequences highlighted in yellow. The cDNA was cloned in the vector pBluescript and T3 and T7 primers were used for sequencing. The cDNA sequence was found to be identical to the corresponding region of contig 1104992. This sequence has been deposited in GenBank under accession number KT601713.

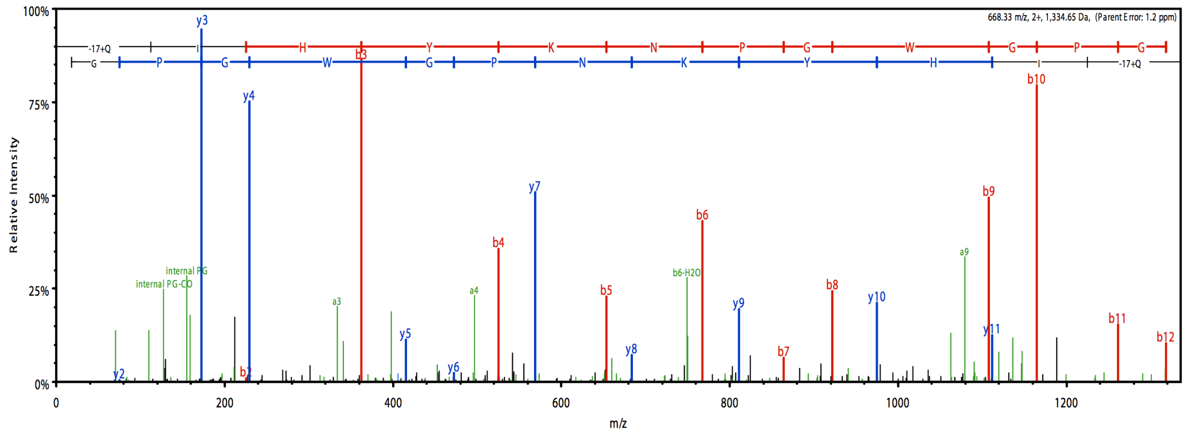

Supplementary Figure S6. Mass spectrometric identification of the neuropeptide ArGnRH (pQIHYKNPGWGPG-NH_2) in an acetic acid extract of radial nerve cords from *Asterias rubens*. The experimentally determined monoisotopic mass of this peptide was 1334.65 Da, consistent with calculated monoisotopic mass (1334.652 Da). The deconvoluted, monoisotopic, singly charged spectrum derived from MS/MS data is shown, with the b series of fragment ions shown in red and fragment ions from the y series shown in blue. Additional identified peptide fragment ions are shown in green, including neutral loss of water.

Mass spectrometry materials and methods, in detail

Frozen radial nerve cords extracts were thawed and an aliquot diluted 10-fold with 0.1% aqueous formic acid, then filtered through a 0.22 µm Costar Spin-X centrifuge tube filter to remove particulates. The extract was analysed by means of nanoflow liquid chromatography with electrospray ionisation quadrupole time-of-flight tandem mass spectrometry (nanoLC-ESI-MS/MS) using a nanoAcquity UPLC system coupled to a Synapt G2 HDMS mass spectrometer (Waters Corporation, Milford, MA, USA) and MassLynx v4.1 SCN 908 software (Waters Corporation, Milford, MA, USA).

The mobile phases used for the chromatographic separation were: 0.1% aqueous formic acid (mobile phase A) and 0.1% formic acid in acetonitrile (mobile phase B). An aliquot containing 15 µL of the extract was applied to a trapping column (Symmetry C18 180 µm × 20 mm, 5 µm particle size, 100 Å pore size, Waters Corporation) using 99.9% mobile phase A at a flow rate of 10 µL min^{-1} for 3 min, after which the fluidic flow path included the analytical capillary column (HSS T3 75 µm×150 mm, 1.8 µm particle size, 100 Å pore size, Waters Corporation). A linear gradient of 5–40% mobile phase B over 105 min was utilized with a total run time of 120 min.

The nanoflow ESI source conditions were as follows: capillary voltage 3.5 kV, sample cone voltage 25 V with a source temperature of 80°C. The instrument was operated in resolution mode (~20,000 measured at full width half height). A data-dependent acquisition was performed that would trigger an MS/MS scan on any multiply charged peptide of intensity ≥ 450 counts/sec within the survey scan *m/z* range 300-1950. A maximum of 5 precursor peptides were selected for MS/MS from each survey scan and MS/MS data collected for 6 scans then combined. Each peptide precursor was then excluded from selection for MS/MS for a period of 20 sec. MS/MS data was collected over *m/z* range 50-1950 using *m/z* and charge state dependent collision energy applied to the trap region.

Tandem mass spectra were extracted by ProteinLynx Global Server version 2.5.1 (Waters Corporation, Milford, MA, USA) with charge state deconvolution and deisotoping performed prior to creation of a peak list file for each sample. All MS/MS samples were

analyzed using Mascot (Matrix Science, London, UK; version 2.5.0). Mascot was set up to search an *Asterias rubens* neuropeptide precursor database containing 168 entries (including ArGnRHP and ArCRZP) assuming non-specific cleavage sites. Mascot was searched with a fragment ion mass tolerance of 0.100 Da and a parent ion tolerance of 25 ppm. Gly-loss+Amide of the C-terminus, gln->pyro-Glu of the N-terminus, amidation of the C-terminus and oxidation of methionine were specified in Mascot as variable modifications.

Scaffold (version Scaffold_4.2.1, Proteome Software Inc., Portland, OR) was used to validate MS/MS based peptide and protein identifications. Peptide Probabilities from Mascot were assigned by the Scaffold Local FDR algorithm. Protein identifications were accepted if they could be established at greater than 99.0% probability and contained at least 1 identified peptide. Protein probabilities were assigned by the Protein Prophet algorithm (Nesvizhskii, Al et al *Anal. Chem*. 2003;75(17):4646-58).

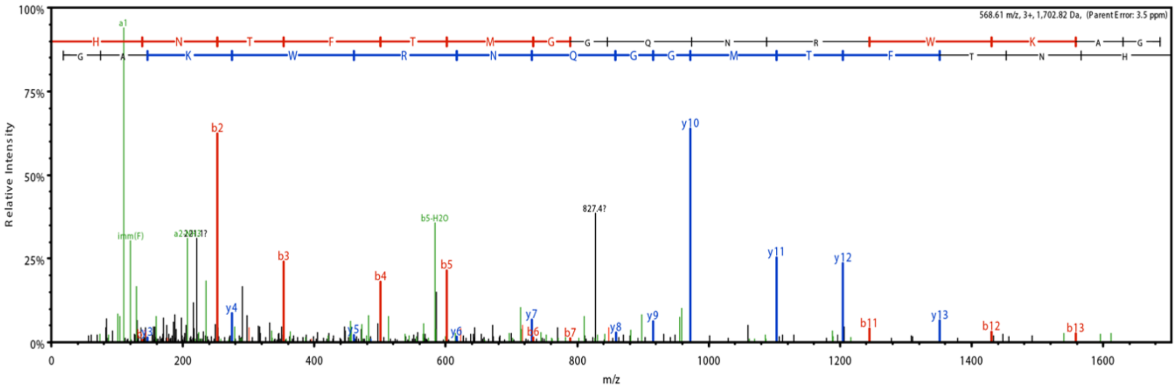

Supplementary Figure S7. Mass spectrometric identification of the neuropeptide ArCRZ (HNTFTMGGQNRWKAG-NH_2) in a methanol - acetic acid extract of radial nerve cords from *Asterias rubens*. The experimentally determined monoisotopic mass of this peptide was 1702.82 Da, consistent with calculated monoisotopic mass (1702.811 Da). The deconvoluted, monoisotopic, singly charged spectrum derived from MS/MS data is shown, with the b series of fragment ions shown in red and fragment ions from the y series shown in blue. Additional identified peptide fragment ions are shown in green, including neutral loss of water and immonium ions.

For mass spectrometry materials and methods in detail, see Fig. S6.

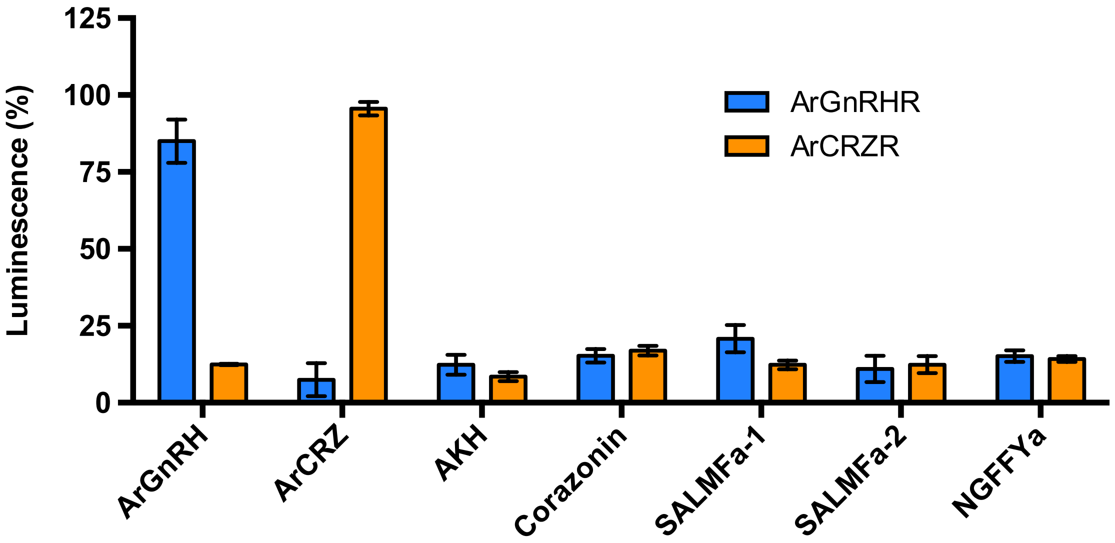

Supplementary Figure S8. ArGnRHR and ArCRZR are specifically activated by ArGnRH and ArCRZ, respectively. Neither ArGnRHR nor ArCRZR are activated by other GnRH/CRZ-type neuropeptides (*Drosophila* AKH; *Drosophila* Corazonin) or by other starfish neuropeptides (SALMFamide-1, SALMFamide-2, and NGFFYamide). All peptides were tested at the concentration of 10^{-5} M and the luminescence responses were normalised to the maximum receptor activation.

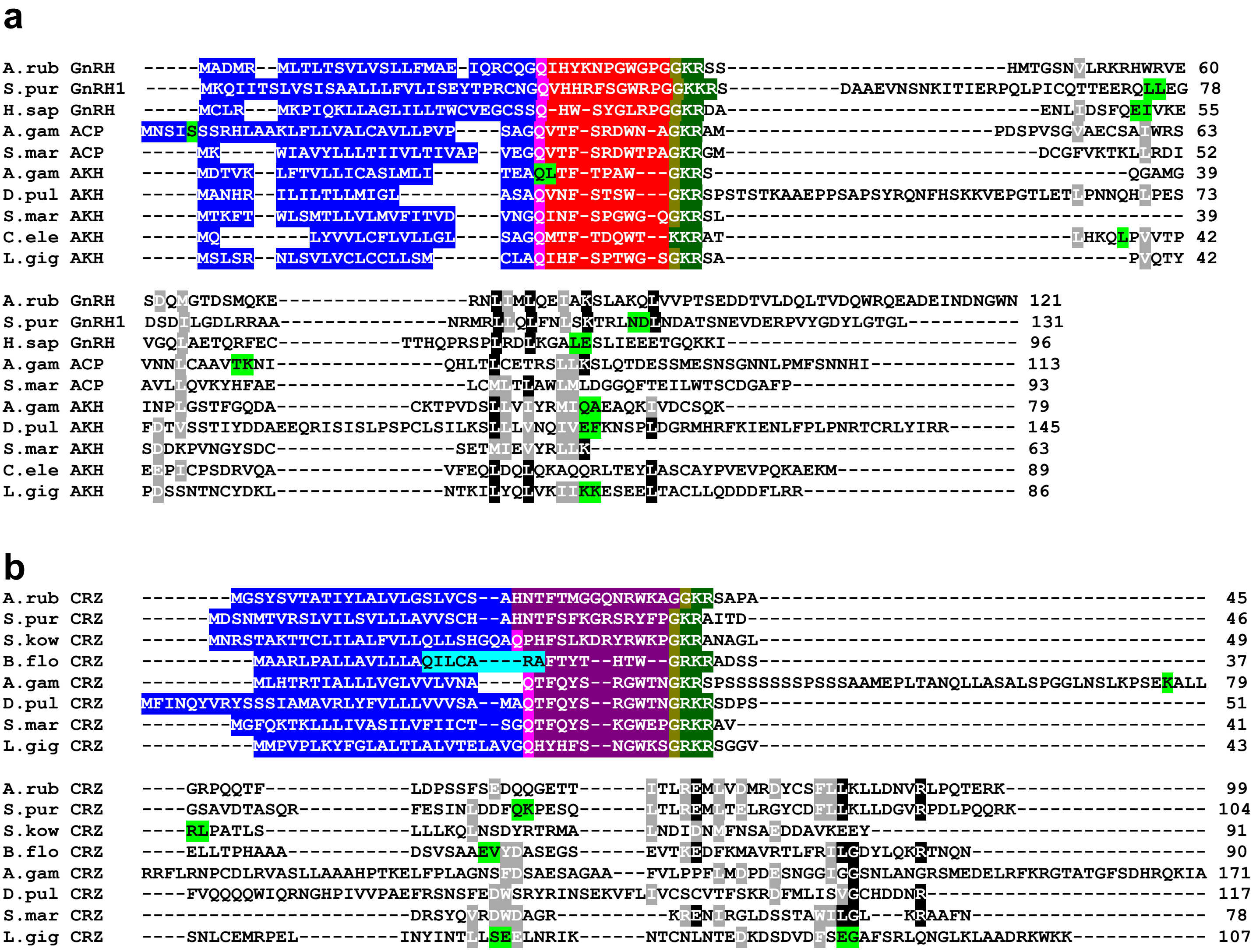

Supplementary Figure S9. Alignments of GnRH/AKH/ACP/CRZ-type neuropeptide precursors. **(a)** Alignment of ArGnRH precursor sequence with GnRH-, AKH- and ACP-type precursors from other species. The predicted or known neuropeptide sequences (without post-translational modifications) are highlighted in red. **(b)** Alignment of ArCRZ precursor sequence with CRZ-type precursors from other species. The predicted or known neuropeptide sequences (without post-translational modifications) are highlighted in purple. For both the alignments, the signal peptides are highlighted in blue, N-terminal glutamine residues that are predicted or known to be converted to a pyroglutamate are highlighted in pink, C-terminal glycine residues that are predicted or known to be substrates for C-terminal amidation are highlighted in olive and di-/tri-basic cleavage sites are highlighted in dark green. The amino acids highlighted in light blue in the *B. floridae* CRZ-type precursor are predicted to be a part of the signal peptide but were proposed previously to form the N-terminal region of the neuropeptide derived from this precursor (Roch, et al. 2014). The positions of introns in genes encoding these proteins are shown by highlighting in light green the amino acid(s) that are interrupted by introns in the corresponding genomic sequence, if this information is available. Note that the deuterostomian GnRH-type precursors (S.pur and H.sap) have two introns, which distinguishes them from deuterostomian CRZ-type precursors (S.pur, S. kow, B.flo) that have only one intron.

Species names are as follows: A.rub, *Asterias rubens*; S.pur, *Strongylocentrotus purpuratus*; H.sap, *Homo sapiens*; A.gam, *Anopheles gambiae*; S.mar, *Strigamia maritima*; D.pul, *Daphnia pulex*; C.ele, *Caenorhabditis elegans*; L.gig, *Lottia gigantea*; B.flo, *Branchiostoma floridae*.

Accession numbers and citations for sequences included in the alignments

a. A. rub GnRH, KT601712, (Semmens, et al. 2016) ; S. pur GnRH1, NW_011972626.1, (Sodergren, et al. 2006); H. sap GnRH, BC126437, (Strausberg, et al. 2002) ; A. gam ACP, XP_563757, (Holt, et al. 2002); S. mar ACP, AFFK01019675, (Chipman, et al. 2014) ; A. gam AKH, XM_001689138, (Holt, et al. 2002) ; D. pul AKH, EFX68649, (Colbourne, et al. 2011); S. mar AKH, AFFK01019834, (Chipman, et al. 2014); C. ele AKH, NM_068369, (Sulson 1998) ; L. gig AKH, FC743844.1, (Simakov, et al. 2013).

b. A. rub CRZ, KT601713, (Semmens, et al. 2016); S. pur CRZ, XR_971124, (Sodergren, et al. 2006) ; S. kow CRZ, XR_438542, (Simakov, et al. 2015); B. flo CRZ, KF601546.1, (Roch, et al. 2014) ; A. gam CRZ, XM_001237037.1, (Holt, et al. 2002) ; D. plu CRZ, EU817501, (Colbourne, et al. 2011) ; S. mar CRZ, AFFK01019339.1, (Chipman, et al. 2014) ; L. gig CRZ, FC805607.1, (Simakov, et al. 2013).

References

Chipman AD, Ferrier DE, Brena C, Qu J, Hughes DS, Schroder R, Torres-Oliva M, Znassi N, Jiang H, Almeida FC, et al. 2014. The first myriapod genome sequence reveals conservative arthropod gene content and genome organisation in the centipede Strigamia maritima. PLoS Biol 12:e1002005.

Colbourne JK, Pfrender ME, Gilbert D, Thomas WK, Tucker A, Oakley TH, Tokishita S, Aerts A, Arnold GJ, Basu MK, et al. 2011. The ecoresponsive genome of Daphnia pulex. Science 331:555-561.

Holt RA, Subramanian GM, Halpern A, Sutton GG, Charlab R, Nusskern DR, Wincker P, Clark AG, Ribeiro JM, Wides R, et al. 2002. The genome sequence of the malaria mosquito Anopheles gambiae. Science 298:129-149.

Roch GJ, Tello JA, Sherwood NM. 2014. At the transition from invertebrates to vertebrates, a novel GnRH-like peptide emerges in amphioxus. Mol Biol Evol 31:765-778.

Semmens DC, Mirabeau O, Moghul I, Pancholi MR, Wurm Y, Elphick MR. 2016. Transcriptomic identification of starfish neuropeptide precursors yields new insights into neuropeptide evolution. Open Biol 6.

Simakov O, Kawashima T, Marletaz F, Jenkins J, Koyanagi R, Mitros T, Hisata K, Bredeson J, Shoguchi E, Gyoja F, et al. 2015. Hemichordate genomes and deuterostome origins. Nature 527:459-465.

Simakov O, Marletaz F, Cho SJ, Edsinger-Gonzales E, Havlak P, Hellsten U, Kuo DH, Larsson T, Lv J, Arendt D, et al. 2013. Insights into bilaterian evolution from three spiralian genomes. Nature 493:526-531.

Sodergren E, Weinstock GM, Davidson EH, Cameron RA, Gibbs RA, Angerer RC, Angerer LM, Arnone MI, Burgess DR, Burke RD, et al. 2006. The genome of the sea urchin Strongylocentrotus purpuratus. Science 314:941-952.

Strausberg RL, Feingold EA, Grouse LH, Derge JG, Klausner RD, Collins FS, Wagner L, Shenmen CM, Schuler GD, Altschul SF, et al. 2002. Generation and initial analysis of more than 15,000 full-length human and mouse cDNA sequences. Proc Natl Acad Sci U S A 99:16899-16903.

Sulson JEaW, R. 1998. Genome sequence of the nematode C. elegans: a platform for investigating biology. Science 282:2012-2018.

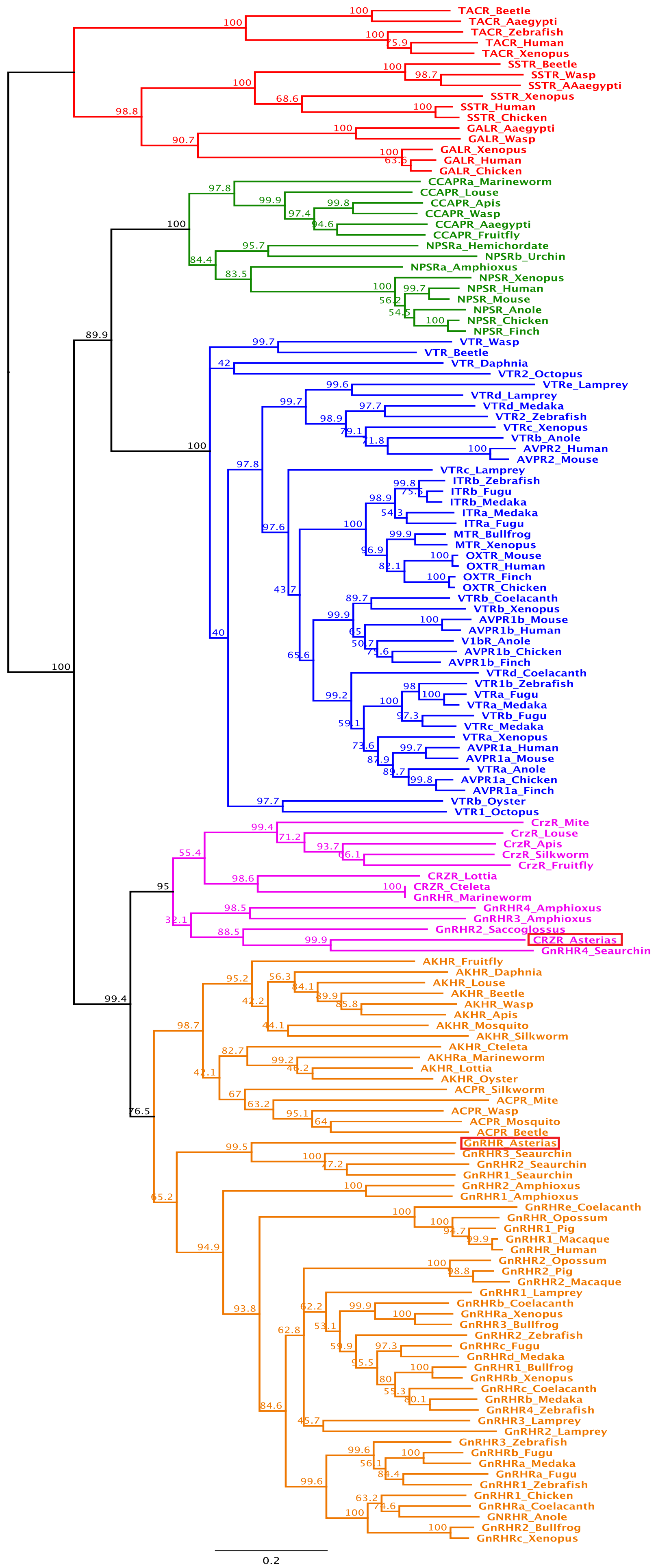

Supplementary Figure S10. **Phylogenetic analysis of GnRH/AKH/ACP-type, corazonin-type, vasopressin/oxytocin-type and NPS/CCAP-typereceptors using a neighbor-joining method reveals distinct clades for each superfamily**. GnRH/AKH/ACP-type receptors are shown in orange, corazonin (CRZ)-type receptors are shown in pink, vasopressin/oxytocin-type receptors are shown in blue and NPS/CCAP-type receptors are shown in green. The outgroup (shown in red) includes tachykinin-type (TACR), somatostatin-type (SSTR), and galanin-type (GALR) receptors. All of the receptor sequences were obtained from Roch et al., 2014, with the exception of the *Asterias rubens* GnRH-type and CRZ-type receptors reported in this paper, which are highlighted (red boxes). The scale bar indicates amino acid substitutions per site. Receptor sequences were aligned and de-gapped as described in Materials and Methods. Geneious 8.0.5 was used to obtain a neighbor-joining tree with 1000 bootstrap replicates.

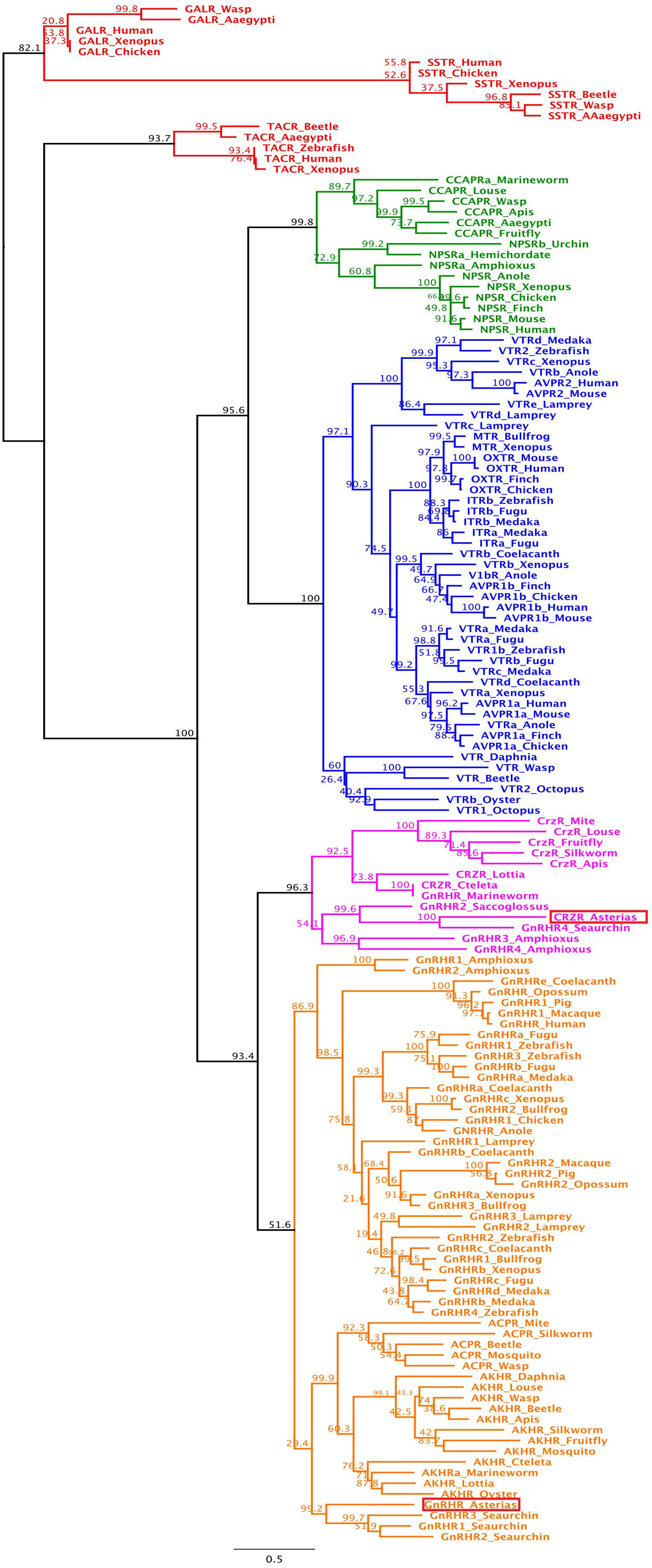

Supplementary Figure S11. Phylogenetic analysis of GnRH/AKH/ACP-type, corazonin-type, vasopressin/oxytocin-type and NPS/CCAP-type receptors using a maximum-likelihood method reveals distinct clades for each superfamily. GnRH/AKH/ACP-type receptors are shown in orange, Corazonin (CRZ)-type receptors are shown in pink, Vasopressin/Oxytocin-type receptors are shown in blue and NPS/CCAP-type receptors are shown in green. The outgroup (shown in red) includes tachykinin-type (TACR), somatostatin-type (SSTR), and galanin-type (GALR) receptors. All of the receptor sequences were obtained from Roch et al., 2014, with the exception of the *Asterias rubens* GnRH-type and CRZ-type receptors reported in this paper, which are highlighted (red boxes). The scale bar indicates amino acid substitutions per site. The analysis was conducted as described in Materials and Methods.
